# Recent advances in the development of antimicrobial peptides against ESKAPE pathogens

**DOI:** 10.1016/j.heliyon.2024.e31958

**Published:** 2024-05-24

**Authors:** Cesar Augusto Roque-Borda, Laura Maria Duran Gleriani Primo, Henrik Franzyk, Paul Robert Hansen, Fernando Rogério Pavan

**Affiliations:** a São Paulo State University (UNESP), Tuberculosis Research Laboratory, School of Pharmaceutical Sciences, Araraquara, Brazil; b Universidad Católica de Santa María, Vicerrectorado de Investigación, Arequipa, Peru; c University of Copenhagen, Faculty of Health and Medical Sciences, Department of Drug Design and Pharmacology, Denmark

## Abstract

Multi-drug resistant ESKAPE pathogens (*Enterococcus faecium*, *Staphylococcus* aureus, *Klebsiella pneumoniae*, *Acinetobacter baumannii*, *Pseudomonas aeruginosa*, and Enterobacter species) are a global health threat. The severity of the problem lies in its impact on mortality, therapeutic limitations, the threat to public health, and the costs associated with managing infections caused by these resistant strains. Effectively addressing this challenge requires innovative approaches to research, the development of new antimicrobials, and more responsible antibiotic use practices globally. Antimicrobial peptides (AMPs) are a part of the innate immune system of all higher organisms. They are short, cationic and amphipathic molecules with broad-spectrum activity. AMPs interact with the negatively charged bacterial membrane. In recent years, AMPs have attracted considerable interest as potential antibiotics. However, AMPs have low bioavailability and short half-lives, which may be circumvented by chemical modification. This review presents recent *in vitro* and *in silico* strategies for the modification of AMPs to improve their stability and application in preclinical experiments.

## 1 Introduction

Multi-drug resistant ESKAPE pathogens (*Enterococcus faecium*, *Staphylococcus* aureus, *Klebsiella pneumoniae*, *Acinetobacter baumannii*, *Pseudomonas aeruginosa*, and Enterobacter species) pose a significant global health problem [1]. Predominantly, infections and fatalities in healthcare settings are attributed to opportunistic bacteria, notably carbapenem-resistant *A. baumannii* and *P. aeruginosa*. Often, inadequate disinfection practices lead to the persistence of strains carrying resistance plasmids, exacerbating diseases and giving rise to frequently incurable co-infections [2].ESKAPE pathogens have been designated priority status by the World Health Organization, each presenting unique resistance profiles, thereby constituting a pivotal concern within the global health landscape [3,4]. These bacteria possess a range of characteristics that make them particularly difficult to combat, including their adeptness at forming biofilms, which enable them to adhere to surfaces and evade antimicrobial treatments effectively [1]. Additionally, they produce various enzymes and toxins that enhance the virulence of the infections they provoke; moreover, their remarkable ability to swiftly adapt to different environments and exchange resistance genes via mechanisms like bacterial conjugation and plasmid transfer further complicates efforts to combat them, altogether creating a significant obstacle in the realm of infectious disease management [5]. Consequently, an array of antimicrobial molecules has undergone extensive testing over the past two decades. While many exhibit commendable antimicrobial activity, a recurring issue arises with suboptimal biocompatibility during *in vivo* biological treatments. As of the latest available data from the Food & Drug Administration as of June 02, 2023, the pharmaceutical landscape remains devoid of newly approved antimicrobial agents. Remarkably, the pharmaceutical approvals of 2023 comprise only combinations of pre-existing drugs, such as vonoprazan/amoxicillin/clarithromycin, primarily employed for *Helicobacter pylori* infections. This prevailing stagnation in the development of novel antimicrobials raises substantial concerns, underscoring the urgent need to address this critical issue (FDA 2023).

In contrast to this situation, multi-drug resistant (MDR) bacteria have been increasing rapidly worldwide and infections caused by these MDR pathogens previously only associated with hospitalized patients have reached a stage where it has now begun spreading among citizens in communities, namely community-acquired infections [6]. This increase in resistance, especially in relation to ESKAPE pathogens, has led to a global initiative two decades ago between the pharmaceutical industry and research groups to search for a new class of antibiotics; as the development of current resistance is associated with clinical treatment failure, additional mortality and healthcare costs [7,8]. Pathogen-directed therapeutics aim to mitigate bacterial toxicity by targeting specific processes altering their virulence factors, while host-directed therapeutics combat superbugs by modulating immune cells, enhancing host cell functions, and modifying disease pathology. The emergence of new antibiotics against global priority superbugs, either entering the market or in clinical development, underscores progress in this field, but ongoing refinement of treatment strategies is essential, considering the potential for resistance development. Rational antibiotic use and adherence to proper hygiene practices among patients are critical in addressing this challenge effectively [9].

Several studies have demonstrated the effectiveness of antimicrobial peptides (AMP) against drug-resistant bacteria [[10], [11], [12], [13]]. They show rapid killing, broad-spectrum antimicrobial activity and are active at micromolar concentrations. AMPs interact with the negatively charged bacterial membrane although intracellular targets are known. In recent years, AMPs have attracted considerable interest as alternative antibiotics [14,15]. The main drawbacks of AMPs are susceptibility to proteases, low bio-availibity and toxicity, which may be circumvented by chemical modification. This review provides recent advances in the design of antimicrobial peptides against ESKAPE bacteria.

### 1.1 ESKAPE bacteria

*Enterococcus faecium*, a globally recognized nosocomial pathogen, has adapted to hospital environments since the 1980s, acquiring resistance to antibiotics. Its successful adaptation in nosocomial settings involves several strategies, including the presence of pathogenicity islands, mobile genetic elements, increased antibiotic resistance genes (e.g., *vanA*), and virulence subproducts (e.g., biofilm formation) [16]. Although *E. faecium* is normally part of the microbiota, it can lead to severe infections in vulnerable individuals, often associated with hospital outbreaks of urinary tract infections, endocarditis, and bacteremia. In this challenging scenario, a significant obstacle in treating enterococcal infections is the emergence of antibiotic resistance in clinical isolates. E. faecium has demonstrated resistance to penicillins, fluoroquinolones, macrolides, and tetracyclines [17,18]. The emergence of vancomycin-resistant strains of *E. faecium* (VREfm) is of significant concern to healthcare professionals due to their high persistence in hospital settings and limited treatment options. Because of its clinical importance and the economic burden on healthcare systems, the WHO has designated VREfm as a high-priority pathogen for drug research [17,18].

*Staphylococcus aureus* colonizes various parts of the human body, including the skin, nose, throat, and intestine, making it an important opportunistic pathogen. This bacterium has the potential to cause severe invasive infections, including bacteraemia, pneumonia, and endocarditis. These infections can manifest as acute, recurrent, or chronic and persistent illnesses [19]. *S. aureus*, one of the few pathogens equipped with numerous immune evasion mechanisms, can secrete molecules that hinder the proliferation of B cells and T cells, impede phagocyte chemotaxis and uptake, inhibit oxidative killing, and block complement activation. This array of strategies enables *S. aureus* to cause recurrent and persistent infections, underscoring the enduring nature of its interaction with the human immune system [20]. This bacterium holds a prominent position among global health concerns owing to its high virulence, remarkable adaptability, and resistance mechanisms against nearly all antimicrobial drugs commonly employed in treatment, including β-lactams, glycopeptides, and oxazolidinones. Notably, strains of vancomycin-resistant *S. aureus* (VRSA) typically harbor the *vanA* operon within the *Tn1546* transposon [21].

*Klebsiella pneumoniae* is a Gram-negative opportunistic pathogen renowned for its significant role in both nosocomial and community-acquired infections, encompassing bacteremia, pneumonia, urinary tract infections, and liver abscesses. This bacterium possesses various accessory genomes of plasmids and chromosome gene loci, which classify its strains into three types: opportunistic, hypervirulent, and MDR. Among these, MDR strains, often associated with antibiotic resistance genes encoded by plasmids and accumulated due to inadequate antibiotic use, pose the most alarming threat [22]. The mainstay treatment for serious *K. pneumoniae* infections, cephalosporin and carbapenem antibiotics, has been compromised by the transfer of genes encoding enzymes such as β-lactamases and carbapenemases. Consequently, the emergence of MDR strains has led to increased mortality rates in *K. pneumoniae* infections, exacerbating the pressing need for effective and safe antimicrobial options [23].

*Acinetobacter baumannii* is a clinically significant bacterial pathogen due to its propensity for causing various infections and its ability to develop resistance to multiple antibiotics, alongside its persistence in hospital environments [24]. Notably, it is a common culprit of nosocomial infections, particularly prevalent in intensive care units, contributing to respiratory tract infections, bloodstream infections, urinary tract infections, and surgical wound infections. The challenge in treating *A. baumannii* infections lies in its resistance to multiple antibiotics, leading to elevated mortality rates, especially among immunocompromised or critically ill patients [24,25]. This bacterium exhibits a remarkable capacity to acquire resistance against a broad spectrum of antibiotics, encompassing β-lactams (*OXA-23*, *OXA-24/40*, *OXA-58* genes), aminoglycosides, quinolones, and carbapenems, necessitating the utilization of last-resort antimicrobial agents [25]. Moreover, its ability to persist in hospital environments for prolonged periods facilitates transmission between patients, thereby fueling nosocomial outbreaks. Consequently, the treatment of *A. baumannii* infections entails significant costs due to the requirement for last-resort antimicrobial agents and prolonged hospital stays [26].

*Pseudomonas aeruginosa* poses significant challenges in healthcare due to its diverse virulence factors and innate resistance mechanisms, causing respiratory, urinary, bloodstream, and wound infections. Its formation of robust biofilms on medical devices and surfaces complicates treatment and contributes to its persistence in hospitals [27]. *P. aeruginosa* possesses multiple efflux pump systems that actively expel antibiotics from within the bacterial cell, reducing their intracellular concentration and efficacy [28].

Genes encoding efflux pumps such as *MexAB-OprM, MexXY, MexCD-OprJ,* and *MexEF-OprN* play crucial roles in mediating resistance to a wide range of antibiotics [29]. Production of beta-lactamases, enzymes that hydrolyze beta-lactam antibiotics such as penicillins and cephalosporins, is a common mechanism of resistance in *P. aeruginosa*, as genes encoding β-lactamases, including AmpC cephalosporinase and extended-spectrum beta-lactamases (ESBLs), enable the bacterium to inactivate these antibiotics [30]. Additionally, alterations in outer membrane porins, such as OprD, can reduce the uptake of antibiotics, particularly carbapenems, into the bacterial cell. Loss or modification of OprD porins limits the effectiveness of carbapenem antibiotics against *P. aeruginosa* [30]. Mutations in genes encoding DNA gyrase (*gyrA* and *gyrB*) and topoisomerase IV (parC and parE) lead to resistance to quinolone antibiotics by diminishing their affinity to their target enzymes. Additionally, *P. aeruginosa* can produce enzymes that chemically modify aminoglycoside antibiotics, thus nullifying their effectiveness [31]. Enzymes such as AAC (6′)-Ib and APH(3′)-IIa phosphorylate and acetylate aminoglycosides, respectively, reducing their ability to bind to bacterial ribosomes [32]. *P. aeruginosa* biofilms provide a protective environment that enhances resistance to antibiotics and host immune defenses, with genes involved in biofilm formation and maintenance, such as those encoding exopolysaccharides and extracellular matrix components, contributing to the bacterium's resilience to antimicrobial agents [33].

*Enterobacter* species pose a formidable challenge within healthcare settings due to their diverse infections and escalating antibiotic resistance. These bacteria are commonly associated with urinary tract infections, bloodstream infections, pneumonia, and wound infections, particularly among hospitalized or immunocompromised individuals [34]. Their ability to form resilient biofilms on medical surfaces further complicates treatment and facilitates their persistence in hospital environments. *Enterobacter* spp. exhibit intrinsic resistance to several antibiotics, including β-lactams and fluoroquinolones, and have increasingly acquired resistance mechanisms, such as ESBLs and carbapenemases [35,36]. This multidrug resistance renders Enterobacter infections difficult to manage and can result in adverse patient outcomes, including treatment failure and heightened mortality rates. Implementing effective infection control protocols and developing novel antimicrobial strategies are imperative to address the rising challenges posed by *Enterobacter* spp. infections in healthcare facilities [36].

ESKAPE pathogens, including *Enterococcus faecium*, *Staphylococcus aureus*, *Klebsiella pneumoniae*, *Acinetobacter baumannii*, *Pseudomonas aeruginosa*, and species of Enterobacter, are of global concern due to their resistance to antimicrobials. The rise in antimicrobial resistance among these pathogens has diminished the effectiveness of treatments for serious infections, leading to increased morbidity and mortality rates. Addressing this pressing issue necessitates coordinated global efforts to surveil antimicrobial resistance. Consequently, there has been a renewed emphasis on developing innovative antimicrobial therapies, improving patient care, and implementing more rigorous stewardship practices.

### 1.2 Antimicrobial peptides

Antimicrobial peptides are a key part of the innate immune system of all multicellular organisms. They are gene-encoded short (10–50 amino acids) cationic and amphipathic molecules with broad-spectrum activity. Furthermore, AMPs have antiviral and immunomodulatory properties [37]. AMPs main mechanism of action is disruption of the bacterial membrane. However other mechanisms are known including interaction with bacterial receptors [38,39], as well as, interfering with DNA, RNA or protein synthesis [[40], [41], [42]].

In recent years, there has been an increasing interest in, antimicrobial peptides as novel antibiotics. However, their main drawbacks are short half-life, toxicity, and high production cost [43]. In this review, we refer to AMPs as specifically active against ESKAPE bacteria (Table 1).

**Table 1:** Selected antimicrobial peptides and analogs with activity against ESKAPE bacteria. The keywords "Antimicrobial peptides" and "ESKAPE bacteria" were employed to gather this information. All data were collected from the Web of Science and Google Scholar databases, accessed in December 2023. The mechanism of action estimated by paper authors were marked by ^2^, the others are determined experimentally. The selection criteria were papers with antimicrobial activity studies of AMPS from 2017–January 2024.

Peptide	Sequence	MIC-values (μg/mL)	Hemolytic activity and cytotoxic	Net Charge	Mechanisms of action	Comments	Ref.
hBD-3	GIINTLQKYYCRVRGGRCAVLSCLPKEEQIGKCSTRGRKCCRRKK	KP: 2–16	Non hemolytic or cytotoxic	+11,7^a^	membrane permeabilization^2^	The results demonstrated excellent activity against ESKAPE bacteria resistant mainly to carbapenems.	[44]
		PA: 2–16					
		AB: 4–16					
Epi-1 22–42	GFIFHIIKGLFHAGKMIHGLV	SA: 8–32	It has hemolytic effects in human erythrocytes at 50 μg/ml.	+2, 7^a^	membrane permeabilization^2^		
		KP: 4–16					
		PA: 4–32					
		AB: 4–32					
PN5	FKFLARTGKFL	SA: 2.65	Non hemolytic or cytotoxic	+3, 7^a^	membrane permeabilization	PN5 showed anti-biofilm activity by carbapenem-resistant *E. coli* and *Staphylococcus aureus*. This peptide has immunomodulatory properties.	[12]
		PA: 5.3					
		AB: 5.3					
		EC: 21.22					
Abarenicin-1 [M9L]	GYCFTACYLRNGVRICYRRCN	SA: 2 μM	Non hemolytic at 32 μM	+4, 7^a^	membrane permeabilization	Abarenicin is a beta- hairpin antimicrobial peptide from the *Abarenicola pacifica;* It has good activity against *E.coli* and *A.baumannii.*	[45]
		KP: 2 μM					
		AB: 0.125 μM					
		PA: 1 μM					
		EC:0.25 μM					
Bip-P-113 Bip: β-(4.4′-biphenyl)alanine	AKR-Bip-Bip-GYKRKFBip	EF: 4	at 25 μg/mL causes cell toxicity (10–20 % cell death and hemolysis)	NR	membrane permeabilization	It is a derivate of P-113, with phenylalanine in their termini, to increase proteolytic stability and salt resistance.	[46]
		SA: 16					
		EC: 32					
Dip-P-113 Dip: β-diphenylalanine	AKR-Dip-Dip-GYKRKF-Dip	EF: 4	at 25 μg/mL causes cell toxicity (10–20 % cell death and hemolysis)	NR	membrane permeabilization		
		SA: 16					
		EC: 64					
Nal-P-113 Nal: β-Naphthylalanine	AKR-Dip-Dip-GYKRKF-Nal	EF: 4	25 μg/mL.	NR	membrane permeabilization		
		SA: 8					
		EC: 32					
PepW	WWWA (Dab)YGL (Dab) LL (Dab)Y (Dab)(Dab)WY	MRSA: 7.8–125	limited cytotoxicity in cell culture	NR	membrane disruption	Adding hydrophobic residues to the sequence decreased the cytotoxic effect.	[47,48]
		KP: 4 μM					
		PA: 62.5–125					
		EC: 1 μM					
Orc1	RRIPFWPPNLPGPRRPPWFLPDFRIPRIPRKR	EF: 16 μM	non-hemolytic	+8, 7^a^	Ativity at the membrane and cytosolic.	The peptide Orc1 permeabilizes the membrane without spectral alteration.	[49,49]
		SA: 32 μM					
		KP: 32 μM					
		AB: 2 μM					
		PA: 32 μM					
		EC: 6 μM					
Del 1	RRIPFWPIPLRWQWPPPWFPPSFPIPRISRKR	EF: 4 μM	hemolytic at 32 μM	+7, 7^a^	membrane permeabilization	It has the translational inhibiting activity and the membranolytic mechanism of action.	
		SA: 8 μM					
		KP: >64 μM					
		AB: 4 μM					
		PA: 16 μM					
		EC:6 μM					
Bal 1	RRIRFRPPRLPRPRPRPWIPPRFPFPRIPGKR	EF: 4 μM	non-hemolytic	+12, 7^a^	inhibitor of protein synthesis	It has a potent antimicrobial effect with a broader spectrum; Its MIC against *E. coli, K.pneumoniae* and *A. baumannii* deserve high light	
		SA: 16 μM					
		KP: 1 μM					
		AB: 1 μM					
		PA: 2 μM					
		EC: 1 μM					
Lip 1	RRIRIRPPRLPRPRPRPWFPPRFPIPRIPGKR	EF: 4 μM	non-hemolytic	+12, 7^a^	inhibitor of protein synthesis	It has a potent antimicrobial effect with a broader spectrum; Its MIC against *E. coli* and *A. baumannii* deserve high light	
		SA: 16 μM					
		KP: 1 μM					
		AB: 1 μM					
		PA: 2 μM					
		EC: 1 μM					
Tur1A	RRIRFRPPYLPRPGRRPRFPPPFPIPRIPRIP	EF: 64 μM	non-hemolytic	+10, 7^a^	inhibitor of protein synthesis	It has a potent antimicrobial effect with a broader spectrum; Its MIC against *E. coli* and *A. baumannii* deserve high light; Not active against *S. aureus*.	
		SA: >64 μM					
		KP: 2 μM					
		AB: 1 μM					
		PA: 16 μM					
		EC: 1 μM					
Tur1B	RRIPFWPPNWPPNWPGPPWLPPWSPPDFRIPRILRKR	EF: 16 μM	non-hemolytic	+6, 7^a^	membrane permeabilization	It has a low charge and high hydrophobicity. It does not have translational inhibiting activity.	
		SA: 32 μM					
		KP: >64 μM					
		AB: 4 μM					
		PA: 32 μM					
		EC: 8 μM					
Bac 7	RRIRPRPPRLPRPRPRPLPFPRPIPRPLPFP	EF: 64 μM	very low cytotoxicity towards animal cells	+10, 7^a^	inhibitor of protein synthesis and membranolytic	It is a bovine peptide with great antimicrobial activity; it can tweak the sequence at the C-terminal.	
		SA: >64 μM					
		KP: 2 μM					
		AB: 2 μM					
		PA: 16 μM					
		EC: 1 μM					
LL37	LLGDFFRKSKEKIGKEFKRIVQRIKDFLRNLVPRTES	EF: 6.25 μM	hemolytic at 170 μM	+6, 7	membrane-targeting bactericidal action	LL37 is a member of cathelicidin family.	[50,51]
		SA: >50 μM					
		KP: 6.25 μM					
		AB: >50 μM					
		PA: >50 μM					
		EC:50 μM					
KR12-NH_2_	KRIVQRIKDFLR-NH_2_	EF: 8	no hemolytic at 0.5–256 μg/mL	+4,7	membrane interaction^2^	KR12 does not cause hemolysis over the whole concentration range (0.5–256 μg/mL).	
		KP: >256					
		AB: 256					
		PA: 16					
C_4_-KR12-NH_2_	C_4_-KR12-NH_2_	EF: 4	no hemolytic activity at 0.5–256 μg/mL	+4,7	membrane interaction^2^	KR12 modified with butyric acid at the N-terminus	
		KP: 128					
		AB: >256					
		PA: 32					
C_6_-KR12-NH_2_	C_6_-KR12-NH_2_	EF: 2	high hemolytic activity.	+4,7	membrane interaction^2^	KR12 modified with hexanoic acid at the N-terminus	
		KP: 16					
		AB: 16					
		PA: 8					
C_8_-KR12-NH_2_	C_8_-KR12-NH_2_	EF: 1	It causes 5 % of hemolysis at 64 μg/mL	+4,7	membrane interaction^2^	KR12 modified with octanoic acid at the N-terminus; it has a great potency against all organisms tested; it was able to eradicate biofilms of *S. aureus.*	
		KP: 2					
		AB: 2					
		PA: 2					
C_10_-KR12-NH_2_	C_10_-KR12-NH_2_	EF: 2	It causes 5 % of hemolysis at 4 μg/mL	+4,7	membrane interaction^2^	KR12modified with decanoic at the N-terminus.	
		KP: 16					
		AB: 8					
		PA: 8					
C_12_-KR12-NH_2_	C_12_-KR12-NH_2_	EF: 4	It causes 5 % of hemolysis at 4 μg/mL	+4,7	membrane interaction^2^	KR12modified with lauric acid at the N-terminus.	
		KP: 32					
		AB: 16					
		PA: 32					
C_14_-KR12-NH_2_	C_14_-KR12-NH_2_	EF: 8	It causes 5 % of hemolysis at 4 μg/mL	+4,7	membrane interaction^2^	It is a derivate of KR12 modified with myristic acid at the N-terminus	
		KP: 64					
		AB: 32					
		PA: 128					
Benzoic acid-KR12-NH_2_	Benzoic acid-KR12-NH_2_	EF: 1	It causes 5 % of hemolysis at 256 μg/mL	+4,7	membrane interaction^2^	KR12 modified with benzoic acid at the N-terminus.	
		KP: 16					
		AB: 16					
		PA: 8					
*trans*-Cinnamic acid-KR12-NH_2_	*trans*-Cinnamic acid-KR12-NH_2_	EF: 1	It causes 5 % of hemolysis at 32 μg/mL	+4,7	membrane interaction^2^	KR12 modified with *trans*-cinnamic acidat the N-terminus.	
		KP: 4					
		AB: 4					
		PA: 4					
17BIPHE2	RIVQRIKDFL	SA: 3.1 μM	hemolytic activity between 150 and 220 μM	+2, 7^a^	membrane permeabilization	Peptide is not a media dependent, with a decrease of LPS neutralization.	[51,52]
		KP: 3.1 μM					
		PA: 6.25 μM					
		EC: 6.25 μM					
FK16	FKRIVQRIKDFLRNLV	SA: 3.1 μM	13.61 ± 3.29 % lysis at 256 μg/mL.	+4, 7^a^	NR	It is a peptide derivate of LL37 without nonessential membrane binding regions.	
		KP: 3.1 μM					
		PA: 25 μM					
		EC: >3.1 μM					
GF17	GFKRIVQKDFLRNLV	SA: 3.1 μM	hemolytic at 180 μM	+3, 7^a^	NR	It is a peptide derivate of LL37 with the most potent activity against resistant *S. aureus*.	
		KP: 3.1 μM					
		PA: 25 μM					
		EC: 6.25 μM					
17tF-W	GX_1_KRVQRKDWRKLV	EF: 3.1 μM	hemolytic at 440 μM	NR	membrane permeation	It is a superior anti-MRSA peptide and showes antibiofilm activity; it shows a high membrane permeation and biofilm disruption.	[53]
		SA: 3.1 μM					
		KP: 12.5 μM					
		AB: 6.25 μM					
		PA: 6.25 μM					
		EC: 3.1 μM					
17 mF-W	GX_1_KRVQRKDWRKLV	EF: 12.5 μM	hemolytic at 440 μM	NR	NR	It is stable in the presence of chymotrypsin, *S. aureus* protease and fungal proteinase K; It is cleaved by trypsin.	
		SA: 12.5 μM					
		KP: 25 μM					
		AB: 6.25 μM					
		PA: 25 μM					
		EC: 6.25 μM					
17W2	GX_1_KRVQRKDWRKLV	EF: 6.25 μM	hemolytic at 440 μM	NR	membrane permeation	It shows a moderate antimicrobial activity.	
		SA: 6.25 μM					
		KP: 6.25 μM					
		AB: 12.5 μM					
		PA: 25 μM					
		EC: 3.1 μM					
17Tf2	GX_1_KRVQRKDWRKLV	EF: 6.25 μM	hemolytic at 440 μM	NR	membrane permeation	It has the preference of an aliphatic portion at position 17 for MRSA killing.	
		SA: 3.1 μM					
		KP: 12.5 μM					
		AB: 3.1 μM					
		PA: 6.25 μM					
		EC: 6.25 μM					
17B-tF	GX_1_KRVQRKDWRKLV	EF: 6.25 μM	hemolytic at 440 μM	NR	membrane permeation	The presence of aromatic rings in this structure is important to kill *K. pneumoniae*.	
		SA: 3.1 μM					
		KP: 12.5 μM					
		AB: 6.25 μM					
		PA: 6.25 μM					
		EC: 6.25 μM					

EF: Enterococcus faecium. SA: *Staphylococcus aureus*. KP: *Klebsiella pneumoniae*. AB: Acinetobacter baumannii. PA: *Pseudomonas aeruginosa*. EC: *Enterobacter cloacae*. NR: No reported.

Mechanism of action estimated by paper authors.

a Not reported by authors, Values calculated with https://pepdraw.com/

### 1.3 Fatty acid modification of antimicrobial peptides

Antimicrobial peptides are modified with fatty acids by chemically linking fatty acid chains to amino acid residues. These modifications occur at different positions along the peptide sequence and may involve adding fatty acids of varying lengths and structures, enhancing the modified peptide's affinity for bacterial cell membranes due to the lipophilic nature of fatty acids [54]. The conjugation of fatty acids to antimicrobial peptides enhances their penetration into bacterial cell membranes, improving antimicrobial effectiveness and conferring stability against enzymatic degradation and denaturation, thereby extending their half-life under physiological conditions and enhancing therapeutic efficacy [55]. This augmentation could result in greater efficacy in lysing bacterial cells, thereby improving infection eradication, while conjugating fatty acids to peptides can potentially decrease the peptides' inherent toxicity; this reduction may enhance their safety for clinical use by lowering the risk of adverse effects [56].

In one study, researchers employed the KR12 (KRIVQRIKDFLR-NH_2_) sequence as a baseline, which demonstrated no antimicrobial activity (128–512 μg/ml) compared to its lipidated counterparts [57]. This suggests that lipidation in these sequences has bolstered their effectiveness against ESKAPE bacteria. The authors noted that the addition of 4-Phenylbenzoic acid, deoxycholic acid or 2-buthyloctanoic acid enhanced antimicrobial activity across all ESKAPE bacteria, with the most notable improvement observed in *E. faecium* (2 μg/ml). A notable aspect of this study was the inclusion of C8 at the C-terminus (C8-KRIVQRIKDFLR-NH_2_) and in the second position (N→C, K(C8)RIVQRIKDFLR-NH_2_), whereas the utilization of C8 at the N-terminus exhibited substantial activity against all ESKAPE bacteria, while the latter completely forfeited its efficacy. The lipidation of AMPs allows improved pharmacokinetic and pharmacodynamic properties, in addition to increasing resistance to unfavorable conditions that can destabilize AMPs [58]. The use of fatty acids is possibly an interesting selective strategy, as it can improve the targeting of several antimicrobial peptides.

According to Harm et al. [59], to design molecules for lipopolysaccharides (LPS) sequestration effectively, some authors propose a crucial division between cationic and apolar domains (amphiphilic feature). C12 is identified as the optimal chain length for LF11-322 (PFWRIRIRR-NH_2_) peptide lipid moiety in antibacterial performance, consistent with other studies. Acyl chain length optimization for LPS binding depends on the AMP, experimental conditions, and LPS source (*P. aeruginosa* vs. *E. coli*). Certain lipid-substituted peptides impact *P. aeruginosa* endotoxin but not *E. coli* LPS, possibly due to variations in lipid A acylation in different bacterial strains. PMB can neutralize both LPS types, while LF11-322 peptides, as per cytokine release assays, don't neutralize endotoxins in serum; only PMB forms a stable LPS-PMB complex in whole blood, reducing proinflammatory mediator release from leukocytes.

A study on ESKAPE bacteria, using ultra-short AMPs (XRR-NH_2_, X = replaced amino acids), found them to be more active when lipidated with C12 and C14 than original sequences. Hemolytic activity was notably impacted when the peptides were conjugated with C14 at concentrations exceeding 25 μg/mL. Conjugation with C12 and C14 resulted in moderate hemolysis. However, when the replaced amino acids were Ala, Cys (Acm), Asp, or Glu, no hemolytic activity was observed. Additionally, they highlighted the use of 2-hexyldecanoic acid as a branched lipid capable of enhancing specificity when incorporated into positively charged AMPs (according to the authors, due to the presence of Arg and Lys) [60]. Supported by another study, the lipidation of the KR12-NH_2_ AMP can enhance its selectivity against bacterial pathogens, particularly by increasing the net charge and utilizing branched carboxylic acids, with the position of the lipidated residue being crucial for its biological activity [57]. Similarly, its activity against *S. aureus* was significantly enhanced when lipidated with C12 at the N-terminus, exhibiting improved efficacy against both planktonic cells and biofilms [50].

Watanabe et al. [61], demonstrated that the palmitic acid (16C) conjugate could selectively target *S. aureus* strains much more compared to lauric acid (12C) conjugates. A study based on anoplin analogs (GLLKRIKTLL-NH_2_) conjugated to fatty acids ranging from 4 to 16 carbons. Initially they showed that the antimicrobial activity was lost when the L-were replaced by a d-amino acid at position 4 or 7; similarly, the minimum inhibitory activity (MIC) values were not improved with other fatty acids, except with the 12C conjugate and in some cases with 10C. These analogs were evaluated in rifampicin- or melittin-resistant *P. aeruginosa* ATCC 27853 and rifampicin-resistant *S. aureus* ATCC (Penicillin MIC-values: >128 μM) and it was concluded that the most active against these pathogens were 12C conjugates (MIC-values: 2–8 μM) [62]. The same authors reported that the application of these conjugates taking advantage of the strategy of dimerization, acetylation and click chemistry [63]. The bactericidal activity of the original peptides showed similar results to those conjugated with 12C (MIC values: 4–16 μM) but curiously the conjugates with other lipids were not very effective against Gram-positive strains such as *S. aureus* and MRSA (MIC values: 8–128 μM and 16–128 μM, Ref: Penicillin <1 and 32 μM, respectively) [63]. Other authors also highlighted the use of C12 and also C8 to provide a better bactericidal activity than the original sequence [64].

A study by Grimsey et al. [58], indicated the relationship of C10 conjugation with C **(**RIRIRWIIR-NH_2_) and D (KRRVRWIIW-NH_2_) peptide activity against MRSA (MIC values 2 and 8 μg/mL) reducing it up to half and 16 times less (1 and 0.5 μg/mL) respectively. In C peptide, its conjugates with C8 and C12 did not show significant difference, however in D peptide, the MIC values of C8 and C12 decreased 4 and 8 times, respectively. This study reinforces the correlation between the number of carbons in the lipid conjugate and the degree of hemolysis exhibited by the AMPs. Specifically, C12 in sequence A resulted in a loss of activity compared to the original sequence. Conversely, in sequence B, C12 enhanced activity twofold against VRE, *E. coli*, and *P. aeruginosa*. While conjugations in MRSA did not yield significant changes, remaining comparable to the original sequence, all conjugations in sequence B (C8–C14) notably enhanced activity. Particularly, C10 demonstrated a 16-fold increase in effectiveness. Moreover, this study emphasizes the importance of incorporating serum, an often overlooked parameter that better simulates bacterial infection. When combined with MRSA, all conjugations rendered the molecules nonviable as MIC levels exceeded 64 μg/mL. These findings underscore the necessity of cautious consideration when transitioning molecules from *in vitro* to *in vivo* phases [58].

An example of peptide lipidation would be polymyxins (cyclic lapidated peptide), which are a group of cationic antibiotics with efficacy against drug-resistant Gram-negative bacteria [65]. Polymyxin B (PMB) and colistin (polymyxin E) are two of the most well-known polymyxins approved by the FDA and have been used in clinical situations to treat serious infections. Its structure consists of a lipid part and a peptide portion, where the lipid part has a long chain of fatty acids, which is essential for antibacterial activity. This lipid part gives them an amphiphilic nature, which allows them to have affinity for both lipid surfaces and aqueous surfaces [66].

Relationship of the conjugated lipid part to the peptide is based on (i) the interaction with cell membranes where the lipid part of polymyxins plays a crucial role in their mechanism of action. When polymyxins come into contact with the cell membrane of Gram-negative bacteria, they insert into the lipid bilayer of the outer membrane. This lipid interaction is essential for membrane permeabilization, leading to disruption of the structural integrity of the bacterial cell. (ii) Membrane permeabilization where insertion of the lipid moiety into the cell membrane increases permeability, leading to leakage of essential intracellular components and ultimately cell death. This mode of action is particularly effective against Gram-negative bacteria, since they have an outer membrane that makes them more resistant to many other antibiotics. (iii) The specificity for Gram-negative bacteria due to its amphiphilic nature allowing the lipid portion to interact with the phospholipids of the outer membrane of Gram-negative bacteria, which are mainly composed of LPS, being more specific. (iv) and overcoming resistance, since the unique mechanism of action is focused on the interaction with the membrane, polymyxins are not affected by many of the common resistance mechanisms [[67], [68], [69]]. However, its clinical utility is primarily restricted to being one of the last resort treatments against multidrug-resistant bacteria, owing to potential adverse effects such as renal and neurotoxicity [70]. Although resistance to polymyxins is rare, efforts are underway to modify these lipopeptides to mitigate side effects and reduce their toxicity [71].On the other hand, Hobby et al. [72], reported that the use of exogenous polyunsaturated fatty acids (PUFAS) as part of the treatment with polymyxin B and colistin, would allow greater bacterial cell permeability; facilitating drug penetration, mainly cationic antimicrobial peptides, since MIC values were reduced even in biofilm-forming strains of *K. pneumoniae* (biofilm-producing bacteria are explained below). These results also indicate that the possible response for a better cell permeability would be related to the position of the unsaturation of the fatty acid, PUFAS with unsaturation closer to –COOH being more permeable. However, a mishandling of PUFAS could lead to an uncontrolled growth of bacteria, since as well as in *K. pneumoniae* and *E. coli*, it was reported that PUFAS were used by these bacteria as a carbon source [72,73]. Likewise, in *E. coli* it was observed that arachidonic and docosahexaenoic acids significantly altered the bacterial phenotype [73]. Therefore, the strategy would be to integrate PUFAS synergistically or conjugated with AMPs for effective treatment.

### 1.4 Cell selectivity of antimicrobial peptides

The cell selectivity of AMPs, critical for therapeutic potential, involves a diverse group of naturally produced molecules integral to innate immune responses against pathogens [74]. The broad-spectrum antimicrobial activity of AMPs, coupled with their ability to rapidly eliminate various ESKAPE pathogens, has generated interest in their development as novel antimicrobial agents [75]. However, a significant challenge in utilizing AMPs therapeutically is their need to distinguish between microbial and host cells to ensure targeted pathogen eradication while minimizing cytotoxic effects on host tissues, emphasizing the importance of achieving optimal cell selectivity to maximize therapeutic efficacy and minimize potential side effects. Understanding the mechanisms governing AMPs' cell selectivity is crucial for their rational design and optimization for clinical use. Disparities in membrane composition, surface charge, and structural characteristics between microbial and host cells contribute to their selective targeting, while dynamic interactions with target membranes, including membrane disruption, pore formation, and intracellular targeting, further modulate their selectivity [76].

The therapeutic index (TI), also known as the safety margin or therapeutic margin, is a pharmacological parameter used to assess the safety and efficacy of a drug. It is calculated by dividing the median lethal dose (LD_50_) by the median effective dose (ED_50_). The LD_50_ represents the dose of a drug that causes death in 50 % of the test animals in a specific study, while the ED_50_ is the dose required to produce the desired therapeutic effect in 50 % of the treated individuals [77]. A higher TI indicates that the drug is safer, as a much higher dose is needed to produce toxic effects compared to the dose required to produce therapeutic effects [78]. Conversely, a lower TI indicates that the drug has a narrower therapeutic margin, meaning there is a higher risk of toxicity relative to the therapeutic dose [79]. Likewise, to consider an AMP as a promising molecule, the MIC-values must be evaluated to eliminate at least 90 % of microorganisms, and the hemolytic activity in human blood cells [80].

Certain authors suggest that the antibacterial effectiveness of peptides is affected by the hydrophobicity of their sequences. They argue that increased hydrophobicity enhances membrane permeability, potentially improving antibacterial activity. However, this structural alteration might compromise peptide selectivity and specificity against bacteria, resulting in heightened cytotoxicity towards human cells [81]. Recently, a study revealed that the activity of the peptide sequence against *S. aureus* and *P. aeruginosa* decreased as they became more polar, with hydrophobicity being directly correlated [82]. Additionally, the authors indicated that during the evaluation of hemolytic activity, significantly relevant parameters such as the effect of albumin are not considered. This is because albumin tends to sequester short chain lipophilic AMPs, resulting in a loss of antimicrobial activity. It is important to highlight that this study involved correlating hemolytic and cytotoxic parameters to extrapolate from *in vitro* to *in vivo* studies. However, it was observed that the AMPs that exhibited the highest cytotoxicity did not demonstrate significant toxicity in mice.

Conditionally the stability of peptides in biological applications can be affected if various concentration parameters similar to biological fluxes are used. For example, the antimicrobial activity evaluated in Muller-Hinton broth (MHB) + 0.9 % NaCl would show different results if fetal bovine serum (FBS) is included; thus, Abarenicin, AA139 and UuBRI-21 showed significantly different results without (0.25 μM) and with FBS (1, 1, 4 μM respectively) [45]. Melittin lost virtually all of its antimicrobial activity when subjected to values above 12.5 % FBS in MHB compared to a RRIRIIIRIRR analog in *E. coli* and combatting drug-resistant fungi [83].

Although this study was not carried out with AMPs and ESKAPE bacteria, it can be seen that bacteria and drugs can have different behavior in more extreme and hostile conditions. Many times, when dealing with intracellular bacteria, it is advisable to include concentrations different from those of the standard study, such as their pharmacokinetic/pharmacodynamic (PK/PD) parameters and/or saline concentration; thus errors during the phase transition from *in vitro* to *in vivo* could be avoided. Therefore, there are still limitations in the studies to determine the MIC values of new drugs [84].

The use of peptides based on toxins or marine substances would be more resistant and stable in hypertonic conditions [45]. It was reported that a peptide modification that includes identical 7-mers would have the ability to resist high salt conditions and proteolytic enzymes such as chymotrypsin, trypsin, and proteinase K. In addition, its selectivity against Gram-negative bacteria was above 99 %, with resistant *P. aeruginosa* as the main objective; concluding that hydrophobicity would be a key factor to improve its stability properties. This report indicated the design of restructured and designed AMPs based on the antitrypsin/antichymotrypsin (XYPX)_n_ sequences where "X" represents Ile, Leu, and Val; and "Y" represents Arg and Lys), and 16 of these analogs obtained significant resistance to enzymatic degradation called antiprotease hydrolytic AMPs [85].

The thermal stability in solution, according to Pohl et al. [86], can be evaluated by classical methods such as differential scanning calorimetry (DSC) that can vary according to the pH to which the peptides are subjected, likewise, its oligomeric and aggregation properties can be identified by dynamic light scattering (DLS) that would be independent of pH. Furthermore, to complement these results, nuclear magnetic resonance (NMR), analytical ultracentrifugation (AUC) and low-angle X-ray diffraction (SAXS) techniques can be combined; this study demonstrated the versatility of the techniques applied to Plectasin. In addition, the RNM 19F, based on the study of fluorinated peptides (section 4) are capable of demonstrating a better understanding, *in vitro* or *in vivo*, between the peptide-membrane interaction, based on the high sensitivity to changes, dispersion of the chemical shift, and low background noise presented by the spin ½ nucleus of the Fluorine probe [87].

### *1.5* *In silico* AMP/drug discovery

Most of the predictions can be generated using platforms that have the 2D AMP structure, however one of the first reports of drug activity prediction based on the 3D structure was successfully launched and applied to AMPs [88]. One of the most widely used AMP repositories is DRAMP, which has a regular DRAMP 2.0 update [89] and DRAMP 3.0 [90]. DRAMP included 22528 entries, 6105 of which are general AMPs (containing natural and synthetic AMPs), 16,110 patent AMPs and 96 AMPs in drug development (preclinical or clinical stage). In the late update, were added 188 stapled antimicrobial peptides belonging to specific AMPs and they are available at http://dramp.cpu-bioinfor.org/(accessed December 26, 2023). We previously reported a list of bacterial genetic targets for drug discovery [37], however, this procedure is accompanied by crystalline protein structures deposited in the Protein Data Bank (PDB), which with the help of mathematical models, molecular docking and online simulations/predictions, can give rise to new specific AMPs against ESKAPE bacteria. For these predictions, a data code of the receptors that can be found mainly on the surface of the bacterial membrane is needed, as well as in other cases, the affinity for blocking some metabolic pathways responsible for the synthesis of the bacterial membrane can be evaluated. In *K. pneumoniae*, the *mrk* gene is responsible for the formation of biofilms due to the presence and modulation of the MrkH protein. On this basis, the AMP-protein interaction and affinity sites of SKITDILAKLGKVLAHV and WYKPAAGHSSYSVGRAAGLLSGLR were evaluated, being potential against MrkH [91]. AMPs can be filtered using an online cytotoxicity and haemolytic activity system such as ToxinPred [92], HemoPred (available at http://codes.bio/hemopred/), which would make it possible to deduce and obtain a reduced range of potentially active AMPs. In biofilm-forming bacteria caused mainly by *A. baumannii, P. aeruginosa, K. pneumoniae*, it is possible to predict and obtain various analogs of a starting molecule using dPABBs [93] or antibacterial activity using PAAS-DBAASP, also for other ESKAPE bacteria [94]. A recently a new platform called sAMPpred-GAT was released based on the information of the predicted peptide structure for AMP recognition and using the structural information, evolutionary profiles and sequence characteristics to build the graphs [95].

**Fig. 1:**
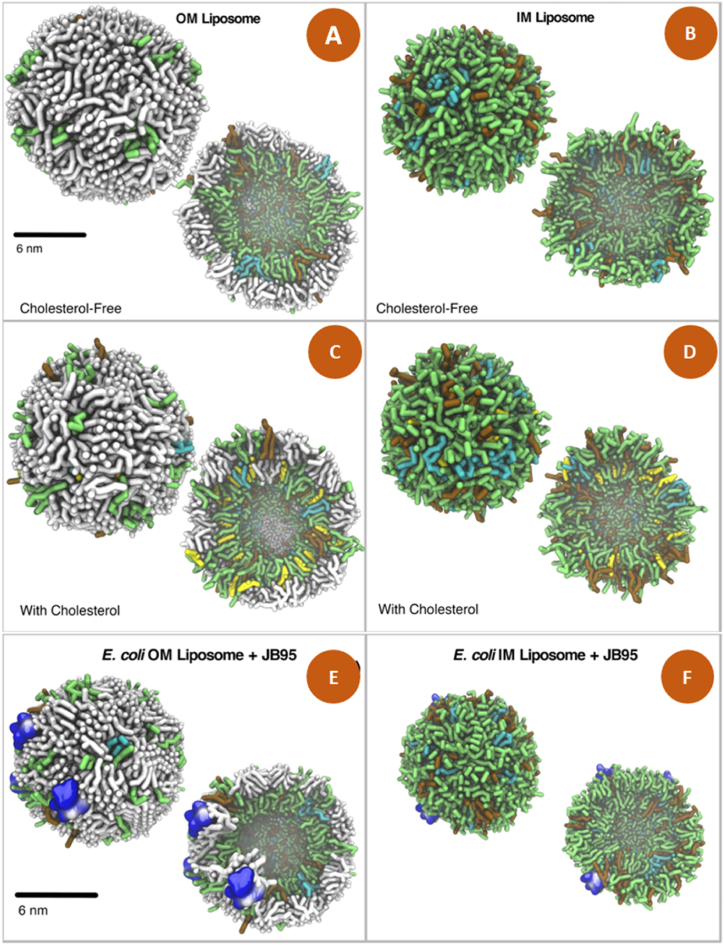
Models of generated *E. coli* liposomes represented as outer membrane (OM) with **(C)** and without cholesterol **(A),** Inner membrane (IM) with **(D)** or without cholesterol **(B).** Interaction between liposomes with cholesterol and the JB95 peptide in OM **(E)** and in IM **(F).** Lipids are represented in sticks where lipid A is in white, 1-Palmitoyl-2-oleoyl-*sn*-glycero-3-phosphatidylethanolamine in green, 1-Palmitoyl-2-oleoyl-*sn*-glycero-3-phosphatidylglycerol in cyan and cardiolipin in grid. Figure reprinted and edited from Franco-Gonzalez et al. [98] Open Access from Scientific Reports by Nature Portfolio 2022. (For interpretation of the references to colour in this figure legend, the reader is referred to the Web version of this article.)

Some peptide-bacteria interaction processes can be predicted using bioinformatics tools, which could help reduce time and money prior to an *in vitro* study. GROMACS simulation package is an important tool for designing more complex molecules such as proteins, lipids and nucleic acids [96,97]. Likewise, a dynamic interaction could generate particles such as vesicles and liposomes that mimic the bacterial cell membrane. A clear example of this interaction is reprinted in Fig. 1 showing *E. coli* liposomes that mimic the intra and extracellular membrane [98]. The authors recommend this strategy against the most problematic Gram-negative bacteria such as *E. coli, K. pneumoniae, A. baumannii,* and *P. aeruginosa* [98].

Likewise, this strategy could be corroborated with studies that determine the mechanism of action of peptides with the interaction of lipids in the membrane using the formation of Large unilamellar vesicles (LUVs) or multilamellar vesicles (MLV) [99]. Many times for the quantification of these, the incorporation of an aromatic amino acid such as Trp is needed, which is used as a fluorescence marker. Alternatively, other molecules have emerged that do not interfere with the structure or its physicochemical properties and antimicrobial activity, such as azulene modified with Ala (β-(1-Azulenyl)-l-Alanine), which, on the contrary, has shown better biocompatibility [[100], [101], [102]].

## 2 Conclusions

Advances in the design of AMPs have opened the way to obtaining new hybrid molecules that have improved the activity of the peptides. Peptidomimetics are still a great world to explore that, in combination with AMPs, would be able to generate potentially viable molecules against the era of resistance. Furthermore, the combined *in silico* and *in vitro* studies give greater robustness to a viable and reliable result. *In silico* and *in vitro* studies complement each other and open the way to better experimental design applied in preclinical studies. Thus, these tools will be useful in the development and discovery of new drugs against the ESKAPE bacteria.

## Funding

Cesar Augusto Roque Borda and Laura Maria Duran Gleriani Primo has a scholarships (#2020/16573-3, #2021/14603-5, #2023/16711-5, #2023/10440-0) and Fernando Rogerio Pavan has a Grant #2023/01664-1 described in the acknowledgments received from the Sat Paulo Research Foundation. The APC was partially paid for by funds from the Sao Paulo State University and partially from the Sao Paulo Research Foundation.

## Ethics declaration

Review and/or approval by an ethics committee as well as informed consent was not required for this study because this literature review only used existing data from published studies and did not involve any direct experimentation/studies on living beings.

## Data availability statement

No data was used for the research described in the article. No data associated in this article has been deposited into a publicly available repository.

## CRediT authorship contribution statement

**Cesar Augusto Roque-Borda:** Writing – review & editing, Writing – original draft, Methodology, Investigation, Funding acquisition, Data curation, Conceptualization. **Laura Maria Duran Gleriani Primo:** Writing – original draft, Investigation. **Henrik Franzyk:** Writing – review & editing, Supervision, Formal analysis. **Paul Robert Hansen:** Writing – review & editing, Supervision, Project administration, Formal analysis. **Fernando Rogério Pavan:** Writing – review & editing, Supervision, Resources, Project administration, Funding acquisition, Conceptualization.

## Declaration of competing interest

The authors declare that they have no known competing financial interests or personal relationships that could have appeared to influence the work reported in this paper.
